# Supplementary material for: Few-Shot Classification of Cryo-EM Micrographs Using Triplet Loss Embeddings
Source: bioRxiv. 2025 Oct 2:2025.10.01.679860. Preprint. [Version 1] doi: 10.1101/2025.10.01.679860 (PMC12621665; doi:10.1101/2025.10.01.679860)
Supplement: 1 [file NIHPP2025.10.01.679860V1-supplement-1.pdf]

bioRxiv preprint doi: https://doi.org/10.1101/2025.10.01.679860; this version posted October 2, 2025. The copyright holder for this preprint (which was not certified by peer review) is the author/funder, who has granted bioRxiv a license to display the preprint in perpetuity. It is made available under aCC-BY 4.0 International license.

A Supplementary Figures and Tables

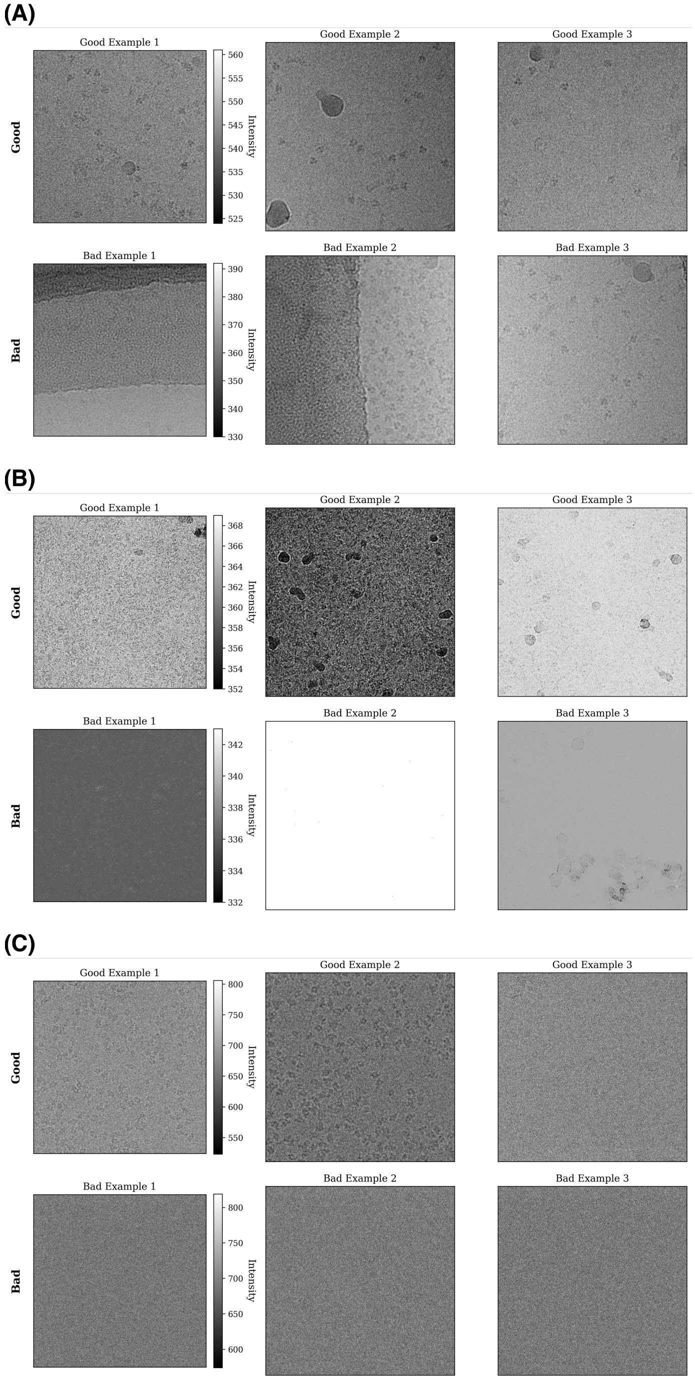

Figure S1: Representative examples of good and bad micrographs from EMPIAR datasets used in this study, showing the variety of defects that must be identified across different samples. For each dataset, top panels show high-quality micrographs suitable for further processing, while bottom panels show micrographs that should be rejected. Bad examples for (A) EMPIAR-10175, exhibiting support film; (B) EMPIAR-10344, showing empty and overexposed images; (C) EMPIAR-10379, showing empty images. All micrographs shown are 4x pixel-binned for improved visual contrast only.

bioRxiv preprint doi: https://doi.org/10.1101/2025.10.01.679860; this version posted October 2, 2025. The copyright holder for this preprint (which was not certified by peer review) is the author/funder, who has granted bioRxiv a license to display the preprint in perpetuity. It is made available under aCC-BY 4.0 International license.

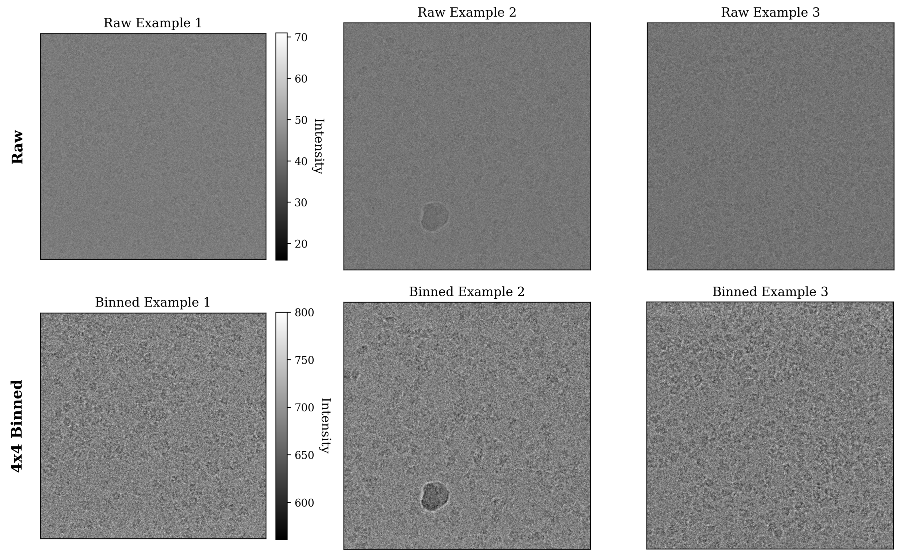

Figure S2: Comparison of raw and binned micrographs from EMPIAR-10379, illustrating the characteristically low SNR of cryo-EM data. Top: Original micrograph at full resolution. Bottom: The same micrograph after 4x pixel binning, showing improved contrast while preserving essential quality indicators. All micrographs shown in Fig. S1 underwent similar binning preprocessing.

Table S1: EMPIAR-10175 Crop-Level Results: AUPRC gain over random ($\mathrm{AUPRC}/p_{\mathrm{pos}}$) versus number of training micrographs per class. Means and standard errors (SE) are aggregated across seeds ($N = 3$).

Init	Shots	Mean gain	SE
ImageNet	1	1.132	0.023
ImageNet	3	1.196	0.012
ImageNet	5	1.181	0.022
ImageNet	10	1.191	0.026
ImageNet	15	1.213	0.029
ImageNet	20	1.181	0.021
ImageNet	25	1.209	0.050
ImageNet	30	1.230	0.020
ImageNet	40	1.228	0.028
ImageNet	50	1.295	0.060
ImageNet	75	1.325	0.048
ImageNet	100	1.283	0.008
Random	1	1.138	0.001
Random	3	1.156	0.004
Random	5	1.156	0.002
Random	10	1.191	0.003
Random	15	1.210	0.012
Random	20	1.131	0.030
Random	25	1.208	0.020
Random	30	1.185	0.015
Random	40	1.224	0.041
Random	50	1.292	0.027
Random	75	1.250	0.020
Random	100	1.301	0.034

bioRxiv preprint doi: https://doi.org/10.1101/2025.10.01.679860; this version posted October 2, 2025. The copyright holder for this preprint (which was not certified by peer review) is the author/funder, who has granted bioRxiv a license to display the preprint in perpetuity. It is made available under aCC-BY 4.0 International license.

Table S2: EMPIAR-10175 Micrograph-Level Results: AUPRC gain over random $(\mathrm{AUPRC}/p_{\mathrm{pos}})$ versus number of training micrographs per class.

Init	Shots	Mean gain	SE
ImageNet	1	1.164	0.028
ImageNet	3	1.225	0.011
ImageNet	5	1.219	0.026
ImageNet	10	1.232	0.047
ImageNet	15	1.237	0.035
ImageNet	20	1.215	0.051
ImageNet	25	1.280	0.070
ImageNet	30	1.314	0.031
ImageNet	40	1.294	0.052
ImageNet	50	1.397	0.094
ImageNet	75	1.445	0.066
ImageNet	100	1.387	0.032
Random	1	1.149	0.000
Random	3	1.157	0.003
Random	5	1.164	0.007
Random	10	1.214	0.003
Random	15	1.234	0.045
Random	20	1.160	0.051
Random	25	1.317	0.028
Random	30	1.207	0.019
Random	40	1.280	0.057
Random	50	1.404	0.030
Random	75	1.352	0.053
Random	100	1.421	0.046

bioRxiv preprint doi: https://doi.org/10.1101/2025.10.01.679860; this version posted October 2, 2025. The copyright holder for this preprint (which was not certified by peer review) is the author/funder, who has granted bioRxiv a license to display the preprint in perpetuity. It is made available under aCC-BY 4.0 International license.

Table S3: EMPIAR-10344 Crop-Level Results: AUPRC gain over random ($\mathrm{AUPRC}/p_{\mathrm{pos}}$) versus number of training micrographs per class.

Init	Shots	Mean gain	SE
ImageNet	1	0.995	0.001
ImageNet	3	1.008	0.010
ImageNet	5	1.062	0.005
ImageNet	10	1.056	0.007
ImageNet	15	1.031	0.009
ImageNet	20	1.047	0.023
ImageNet	25	1.044	0.018
ImageNet	30	1.080	0.003
ImageNet	40	1.072	0.018
ImageNet	50	1.078	0.005
ImageNet	75	1.073	0.006
ImageNet	100	1.073	0.010
Random	1	0.986	0.002
Random	3	0.988	0.002
Random	5	1.023	0.004
Random	10	1.050	0.011
Random	15	1.049	0.004
Random	20	1.040	0.003
Random	25	1.049	0.006
Random	30	1.071	0.010
Random	40	1.067	0.003
Random	50	1.063	0.002
Random	75	1.078	0.006
Random	100	1.097	0.004

bioRxiv preprint doi: https://doi.org/10.1101/2025.10.01.679860; this version posted October 2, 2025. The copyright holder for this preprint (which was not certified by peer review) is the author/funder, who has granted bioRxiv a license to display the preprint in perpetuity. It is made available under aCC-BY 4.0 International license.

Table S4: EMPIAR-10344 Micrograph-Level Results: AUPRC gain over random $(\mathrm{AUPRC}/p_{\mathrm{pos}})$ versus number of training micrographs per class.

Init	Shots	Mean gain	SE
ImageNet	1	0.994	0.000
ImageNet	3	1.008	0.012
ImageNet	5	1.070	0.006
ImageNet	10	1.063	0.007
ImageNet	15	1.039	0.011
ImageNet	20	1.049	0.024
ImageNet	25	1.047	0.020
ImageNet	30	1.083	0.003
ImageNet	40	1.079	0.019
ImageNet	50	1.084	0.006
ImageNet	75	1.081	0.005
ImageNet	100	1.080	0.012
Random	1	0.986	0.003
Random	3	0.986	0.002
Random	5	1.028	0.006
Random	10	1.055	0.011
Random	15	1.049	0.006
Random	20	1.041	0.004
Random	25	1.051	0.007
Random	30	1.075	0.009
Random	40	1.069	0.004
Random	50	1.066	0.004
Random	75	1.083	0.005
Random	100	1.099	0.003

bioRxiv preprint doi: https://doi.org/10.1101/2025.10.01.679860; this version posted October 2, 2025. The copyright holder for this preprint (which was not certified by peer review) is the author/funder, who has granted bioRxiv a license to display the preprint in perpetuity. It is made available under aCC-BY 4.0 International license.

Table S5: EMPIAR-10379 Crop-Level Results: AUPRC gain over random ($\mathrm{AUPRC}/p_{\mathrm{pos}}$) versus number of training micrographs per class.

Init	Shots	Mean gain	SE
ImageNet	1	1.224	0.005
ImageNet	3	1.233	0.000
ImageNet	5	1.233	0.000
ImageNet	10	1.235	0.003
ImageNet	15	1.238	0.004
ImageNet	20	1.231	0.001
ImageNet	25	1.233	0.000
ImageNet	30	1.232	0.001
ImageNet	40	1.239	0.003
ImageNet	50	1.245	0.001
ImageNet	75	1.246	0.001
ImageNet	100	1.244	0.001
Random	1	1.220	0.000
Random	3	1.232	0.001
Random	5	1.232	0.001
Random	10	1.230	0.000
Random	15	1.231	0.001
Random	20	1.231	0.000
Random	25	1.231	0.000
Random	30	1.232	0.000
Random	40	1.239	0.003
Random	50	1.234	0.002
Random	75	1.244	0.001
Random	100	1.238	0.002

bioRxiv preprint doi: https://doi.org/10.1101/2025.10.01.679860; this version posted October 2, 2025. The copyright holder for this preprint (which was not certified by peer review) is the author/funder, who has granted bioRxiv a license to display the preprint in perpetuity. It is made available under aCC-BY 4.0 International license.

Table S6: EMPIAR-10379 Micrograph-Level Results: AUPRC gain over random $(\mathrm{AUPRC}/p_{\mathrm{pos}})$ versus number of training micrographs per class.

Init	Shots	Mean gain	SE
ImageNet	1	1.224	0.005
ImageNet	3	1.233	0.000
ImageNet	5	1.233	0.000
ImageNet	10	1.237	0.005
ImageNet	15	1.241	0.005
ImageNet	20	1.238	0.004
ImageNet	25	1.231	0.000
ImageNet	30	1.233	0.000
ImageNet	40	1.247	0.000
ImageNet	50	1.247	0.000
ImageNet	75	1.247	0.000
ImageNet	100	1.247	0.000
Random	1	1.220	0.000
Random	3	1.233	0.000
Random	5	1.233	0.000
Random	10	1.227	0.000
Random	15	1.229	0.001
Random	20	1.229	0.000
Random	25	1.229	0.000
Random	30	1.229	0.001
Random	40	1.242	0.006
Random	50	1.236	0.005
Random	75	1.247	0.000
Random	100	1.247	0.000

Notes: Random baseline for AUPRC equals the positive-class prevalence p_{pos}. Gains were computed per run as AUPRC/p_{pos} using the confusion matrix to estimate p_{pos} at each level, then averaged across seeds.
